# Closed-loop versus open-loop “remind-to-move” treatment using wearables for hemiparetic upper extremity in patients after stroke: A proof-of-concept study

**DOI:** 10.1017/wtc.2025.10017

**Published:** 2025-07-17

**Authors:** Kenneth N. K. Fong, Jasmine P. Y. Pak, Alissa H. L. Koo, Maggie M. K. Szeto, Natalie M. T. Wong, Keily K. Y. Yau, Sharon F. M. Toh, Vivian W. Lou, Hector W. H. Tsang, Gary K. K. Lau

**Affiliations:** 1 Department of Rehabilitation Sciences, The Hong Kong Polytechnic University, Hong Kong SAR; 2 Research Centre for Assistive Technology, The Hong Kong Polytechnic University, Hong Kong SAR; 3 Singapore Institute of Technology, Singapore, Singapore; 4 Department of Social Work and Social Administration, The University of Hong Kong, Hong Kong SAR; 5 Division of Neurology, Department of Medicine, The University of Hong Kong, Hong Kong SAR

**Keywords:** wearables, open-loop, closed-loop, upper limb, learned nonuse, stroke

## Abstract

This is a proof-of-concept study to compare the effects of a 2-week program of “Remind-to-move” (RTM) treatment using closed-loop and open-loop wearables for hemiparetic upper extremity in patients with chronic stroke in the community. The RTM open-loop wearable device has been proven in our previous studies to be useful to address the learned nonuse phenomenon of the hemiparetic upper extremity. A closed-loop RTM wearable device, which emits reminding cues according to actual arm use, was developed in this study. A convenience sample of 16 participants with chronic unilateral stroke recruited in the community was engaged in repetitive upper extremity task-specific practice for 2 weeks while wearing either a closed-loop or an open-loop ambulatory RTM wearable device on their affected hand for 3 hrs a day. Evaluations were conducted at pre-/post-intervention and follow-up after 4 weeks using upper extremity motor performance behavioral measures, actual arm use questionnaire, and the kinematic data obtained from the device. Results showed that both open-loop and closed-loop training groups achieved significant gains in all measures at posttest and follow-up evaluations. The closed-loop group showed a more significant improvement in movement frequency, hand functions, and actual arm use than did the open-loop group. Our findings supported the use of closed-loop wearables, which showed greater effects in terms of promoting the hand use of the hemiparetic upper extremity than open-loop wearables among patients with chronic stroke.

## 1. Background

Stroke is a “focal (or at times global) neurological impairment of sudden onset, and lasting more than 24 hrs (or leading to death), and of presumed vascular origin” (World Health Organization, 2006, 1–4). There is a wide range of symptoms following stroke onset, including hemiplegia over the contralateral side of the damaged cortical area.

Upper extremity hemiplegia is one of the most widely known impairments resulting from stroke; 80% of stroke patients experience upper limb motor dysfunctions (Ingram et al., 2021), which is a major contributor to their loss of independence (Faria-Fortini et al., 2011), with only around 5% of patients demonstrating complete functional recovery 6 months after onset (Kwakkel et al., 2003). Hemiparetic upper limb impairment is characterized by a unilateral loss of movement, coordination, sensation, and dexterity (Rodgers et al., 2019). These manifestations are brought about by muscle weakness or paresis, spasticity, and abnormal motor synergies, as well as chronic pain (Raghavan, 2015). As a result, stroke patients typically exhibit abnormal compensatory movements and a reduction in the use of the hemiparetic arm, leading to difficulties performing activities of daily living (ADLs) such as dressing and grooming. With the recent advances in technology, the use of wearables is growing in the field of research in stroke rehabilitation. Apart from kinematic measurements such as using an accelerometer to measure step counts, some wearables can now provide augmented feedback, which can be used to guide patients in their daily self-directed training; such feedback from the wearables makes them a user-friendly tool for physical rehabilitation interventions for people with stroke, beyond just their measurement capabilities (Toh et al., 2023a). According to our recent review on home-based wearable technologies in physical rehabilitation for stroke, most of the studies focus on the upper rather than the lower extremities. Among them, stimulation-based and activity trackers are of strong evidence and moderate evidence, respectively (Toh et al., 2023b).

Among the upper limb wearables, “Remind-to-move” (RTM) has been developed to address the learned nonuse phenomenon of hemiparetic upper extremity (Fong et al., 2011). Learned nonuse in upper limb function is one of the long-term behavioral consequences of hemiparetic upper limb impairment (Raghavan, 2015). This problem is caused by the suppression of movement, primarily due to unsuccessful motor attempts in patients’ more-affected upper extremities, rather than due to weakness or sensory loss resulting from damage to brain cells (Taub et al., 2006). Patients tend to rely on their less-affected upper extremities to perform daily activities. However, decreased movement of more-affected upper extremities further increases the chance of unsuccessful motor attempts, which creates a vicious cycle. As time progresses, the nonuse of affected upper extremities becomes a habitual issue (Bailey et al., 2015). Even if patients are capable of moving the affected side, they reduce the frequency with which they incorporate the affected side into functional activities (Taub et al., 1993). As a result, patients may encounter difficulties performing activities of daily living, particularly bilateral tasks, with the use of one hand. Different treatment modalities have been developed in order to overcome the learned nonuse issue, such as constraint-induced movement therapy (CIMT) – a level of A in evidence treatment. CIMT has been used to increase the use of affected upper extremities through massed intensive upper limb practice in 2 weeks, with the patients’ unaffected upper extremities restrained for 5 hrs per day (Fritz et al., 2005).

Different from CIMT, RTM does not involve the restraint of the unaffected upper extremities; instead, the stroke patients are reminded to use their affected upper extremities more frequently in daily life (Fong et al., 2011). They wear a sensory cueing wristwatch on their paretic arm to increase their awareness of moving the affected upper limb. RTM is a completely new concept in physical rehabilitation. In our previous paper on children with cerebral palsy, we found that RTM demonstrated therapeutic effects equivalent to those of CIMT in manual dexterity and functional hand use (Dong et al., 2017). In our randomized controlled trial, we found that RTM could promote more arm recovery than the sham or control could, and, hence, it produced an optimal functional improvement for subacute stroke patients (Wei et al., 2019a). Another of our recently published papers presented an examination of the neural mechanism of RTM using functional near-infrared spectroscopic topography (fNIRS) (Bai and Fong, 2020). We found that RTM was beneficial to stroke patients in that it elicited a higher level of activation than the sham did in the contralateral somatosensory association cortex, primary motor cortex, primary somatosensory cortex, and dorsolateral prefrontal cortex, in both the healthy and the stroke participants. Our imaging study shows that RTM enhances the recruitment of the contralateral primary motor cortex, and that effect appears to be associated with increased attention allocation toward moving the arms upon sensory cueing in the form of vibration (Bai and Fong, 2020). In our recent randomized clinical trial, RTM using a smart reminder wearable, compared with a sham device, has been proven to be potentially efficacious in improving upper limb impairment of the hemiparetic upper extremity after stroke in home-based telerehabilitation through the use of smartphones (Toh et al., 2025).

In the current study, to investigate the proof-of-concept for which types of cueing mechanisms of upper limb wearable might be useful in human response, two types of RTM treatments have been developed by the team: the open-loop RTM and the closed-loop RTM. These two types of treatment are based on the difference between open-loop and closed-loop movement theories. Open-loop and closed-loop systems originate from control theory in physics (Antony et al., 2022). Both are used to produce desirable effects in a given environment. An open-loop circuit does not consider the current state of the environment, while a closed-loop circuit monitors the change in the environment continuously. The timing of the intervention in a closed-loop system is determined by the feedback mechanism. Open-loop and closed-loop systems have been applied in various treatments and rehabilitation programs. According to one study, closed-loop neuromodulation is clinically more effective than open-loop neuromodulation in the treatment of pain, epilepsy, and Parkinson’s Disease (Mirza et al., 2019).

In the open-loop RTM, the wristwatch provides sensory cues to patients at a fixed time interval through vibrations. The wearer who wants to stop the vibration must press the acknowledge button on the top of the device as soon as possible, otherwise it will be activated continuously as long as the button is not pressed (Fong et al., 2011). As the external sensory signals are directed at patients’ affected hands, they can effectively promote their attention over the hemiparetic upper extremity; hence, patients are encouraged to increase the frequency of movement of their paretic upper extremity in daily functioning (Fong et al., 2013). Previous findings have shown that open-loop RTM is useful in enhancing the motor performance of affected upper extremities in patients with chronic stroke by incorporating sensory cueing and limb activation (Fong et al., 2011). To further enhance the effectiveness of open-loop RTM, closed-loop RTM has been developed to encourage patients to take the initiative to perform more movements involving their hemiparetic arm. In closed-loop RTM, in order to close the feedback loop, the frequency of reminders is inversely related to the usage of the hemiparetic arm. The patients will be reminded less frequently when they move their affected upper extremities more frequently. Theoretically, the closed-loop version is more effective than the open-loop version in terms of habit internalization and the retention time in regard to new motor patterns learned by patients. However, to the best of our knowledge, no prior research has been conducted to investigate the effects of closed-loop versus open-loop RTM for hemiparetic upper extremity recovery in patients with stroke specifically.

The purpose of this study is to investigate and compare the effects of the RTM treatment using closed-loop versus open-loop wearables in the context of promoting stroke patients’ hemiparetic upper extremity functioning. We hypothesize that the closed-loop training will demonstrate more significant improvements in terms of the frequency and quality of movements than will the open-loop training using the upper limb wearables.

## 2. Methods

### 2.1. Participants

A randomized controlled pilot trial was adopted for this study. A total of 16 community residents with chronic unilateral stroke were recruited by convenience sampling from stroke self-help groups in Hong Kong. Inclusion criteria were: (1) hemorrhagic or ischemic stroke; (2) stroke involving the unilateral hemisphere; (3) aged 18 years or above; (4) chronic stroke with onset over 6 months before the study; (5) a functional level of the hemiparetic upper limb, as indicated by the Functional Test for the Hemiplegic Upper Extremity (FTHUE), level ≥ 3 (Fong et al., 2004); (6) able to understand and follow verbal instructions; (7) no simultaneous participation in studies related to the brain and upper limb; and (8) able to understand the meaning of the study and provide informed consent to participate. The inclusion criterion of chronic stroke with an onset of more than 6 months is supported by the concept of “slowed-down spontaneous recovery,” to allow a higher potential in regard to brain reorganization responsivity to the interventions in this study (Grefkes and Fink, 2020, 17). To understand which level of severity of hemiparetic upper extremity functioning would benefit most from the treatment, participants were stratified further into lower functioning (FTHUE levels of 3–4) or higher functioning groups (FTHUE levels ≥5) (Fong et al., 2011). Potential participants were excluded if they: (1) have a history of other neurological diseases, including but not limited to Parkinson’s disease; (2) received oral or injective Botox within 6 months before the study; (3) excessive paretic limb spasticity, measured using a score ≥2 for the Modified Ashworth Scale (Bohannon and Smith, 1987).

Participants were randomly assigned to two groups: either a closed-loop training group or an open-loop training group. Written informed consent was obtained from all participants prior to the start of the study. The study was carried out in accordance with the principles of the Declaration of Helsinki. Ethical approval was obtained from the Hong Kong Polytechnic University (Ref. no.: HSEARS20200907005).

### 2.2. Wearable device with open-loop and closed-loop versions

**Figure 1.**
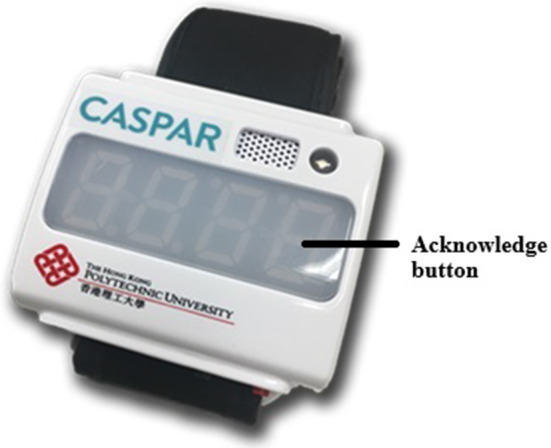
Wristwatch device with an acknowledge button that lights up and vibrates to remind the user to move the hemiparetic upper extremity.

The sensory cueing wristwatch (SCW-V2) is designed to provide pertinent electronic sensory signals to hemiplegic patients, in order to raise their awareness of the hemiparetic limb, as in previous studies (Figure 1) (Fong et al., 2011; Wei et al., 2019a) (Figure 1). The wristwatch is small and lightweight (78 g), with nonallergenic neoprene straps that can be fastened with Velcro for easy anchor onto the wrist. An acknowledge button is built into both versions that users can use to stop the vibration. The wristwatch has a built-in accelerometer to detect the amount of arm movement in the X, Y, and Z directions, as well as the reaction times in terms of the user’s stopping the cue (Fong et al., 2011). In this study, acceleration was sampled at 5 Hz, and a 2-s recording epoch time was used (Fong et al., 2011).

**Figure 2.**
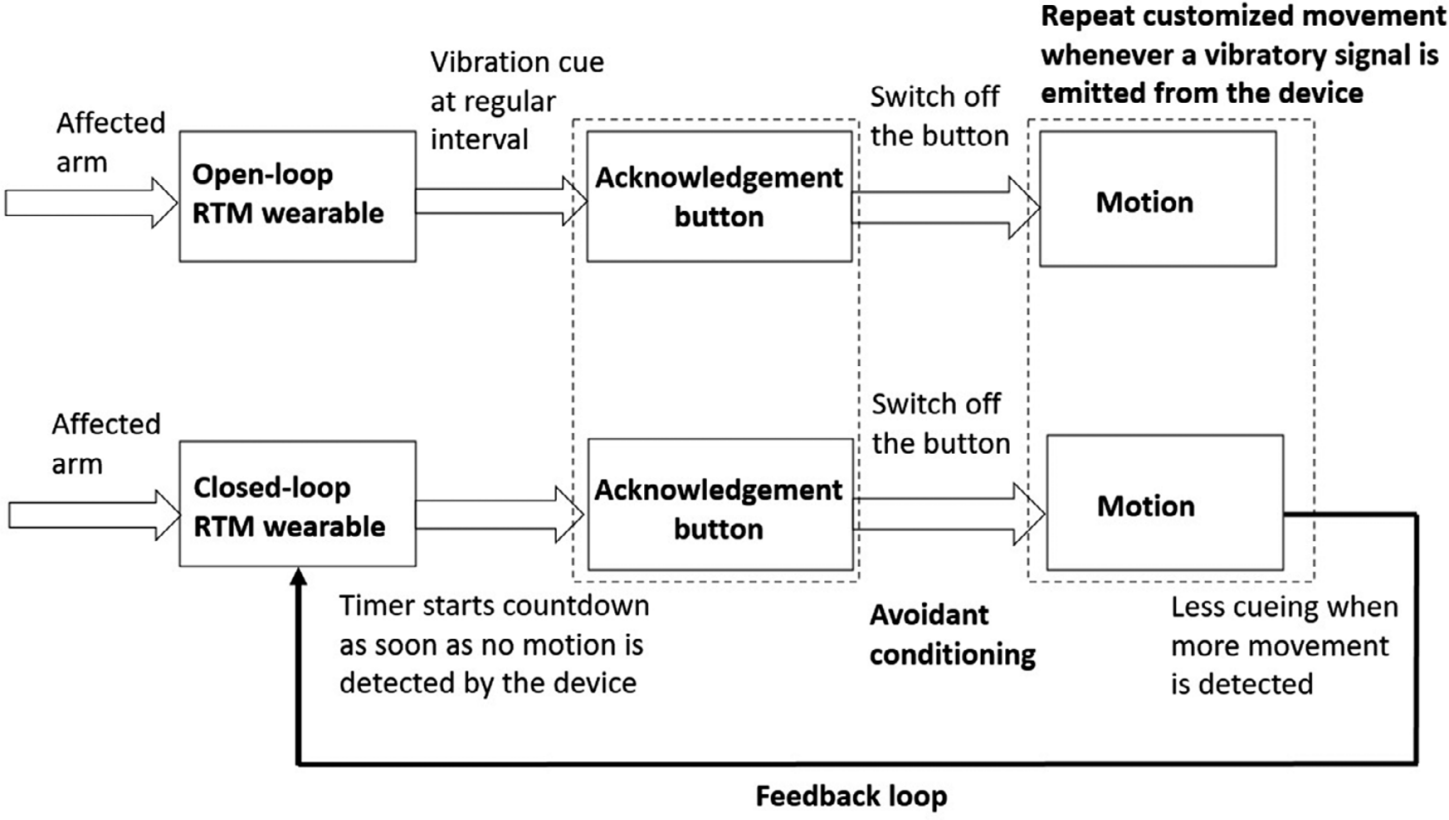
Mechanism of open-loop and closed-loop RTM wearables.

In this study, we have developed two research versions of the device with identical outlook (Figure 1). In the open-loop version, a vibration cue (196 Hz, similar to the vibration mode of a smartphone) with a 2-s on/off cueing pattern is set to occur at 10-min intervals. Built into the device is an acknowledgement button that is used to stop the vibration cue and that will be activated continuously as long as the button is not pressed (Fong et al., 2011). In the closed-loop version, a vibration cue of the same frequency and cueing pattern is set to vibrate irregularly as long as no movement of the hemiparetic arm is detected by the built-in accelerometer, which starts counting down as soon as no movement is detected by the device. We hypothesized that arms are at rest (without movement) for approximately 50% of waking hours, this implies they are actively moving for approximately 50% of the time (excludes walking activity); however, this figure varies widely in terms of frequency and intensity of arm movements depending on factors such as occupation, lifestyle, daily tasks, and so forth, as well as the handedness and impairment levels of the hemiparetic upper extremity. In our previous study of comparing the differences in activity count on a series of bilateral and specific daily tasks for the upper extremities of patients after stroke, the movement ratio between the affected and unaffected upper extremities was approximately one-third for those with lower functioning (FTHUE levels of 3–4) and two-third for those with higher functioning groups (FTHUE levels ≥5) (Wei et al., 2019a). Therefore, the default mode for the countdown mechanism in the closed-loop version was preset at either 2-min or 5-min customized to lower or higher upper extremity functioning, respectively, in order to be comparable to the 10-min intervals in the open-loop version. No movement from the hemiparetic upper limb detected by the device during this period triggers the timer to count down again. Based on the avoidant conditioning theory, there will be less cueing when more movement is detected; hence, this can promote more movement and hand use from the hemiparetic upper extremity. Since this study is for proof-of-concept, both versions are not connected to the smartphone; instead, they are driven by a program stored at the mini-SD card inside the device. The algorithms of both open-loop and closed-loop versions are summarized in Figure 2. Although the cueing mechanisms for both versions are different, to make it comparable in the study, they are of the same outlook, size, and vibration frequency in the cueing.

### 2.3. Procedures

Participants were instructed to wear the device on their affected arm for a total of three waking hours per day during a 2-week period, with a fixed daily schedule, while simultaneously performing repetitive individualized tasks with the same arm. A 2-hrs baseline assessment and treatment session was conducted for each participant, to enable them to familiarize themselves with the tasks and operation of the wristwatch device. Tailored exercises for the affected arm were prescribed to each participant, according to the severity of impairment as reflected by the FTHUE. Participants were asked to repeat five sets of tasks five times whenever a vibratory signal was emitted from the wristwatch. During training, customized tasks were tailored to participants according to their arm impairment levels, with reference to our previous study; they were repetitive movements that targeted the improvement of a range of motion, strength, endurance, and fine motor tasks (Fong et al., 2011). In the open-loop group, participants were told to self-initiate the customized tasks, and the vibration cues would come every 10 mins regularly to remind them to do so (Wei et al., 2019a), whereas participants in the closed-loop group were told to self-initiate similar exercise, and the device would cue them when they were not moving or exercising enough. A document with the exercise regimen written out and illustrated with pictures was sent to each participant to facilitate their practice at home. Participants were also encouraged to move their arms outside the 3-hrs period, even when no signals were emitted. One intermediate visit by the investigators was arranged after 7 days to collect kinematic data from the devices, as well as participants’ verbal feedback on using the wristwatch and their perceived task performance. During this visit, compliance was monitored along with the attainment of target movements during the practice of the assigned tasks. The task difficulty was modulated by the investigators according to users’ experiences at this point.

### 2.4. Measurements

Information about demographic characteristics, medical history, the FTHUE level, the MAS score, and the self-perceived percentage of sensation in the hemiparetic upper extremity of participants was collected (Fong et al., 2011). Three primary outcome measures were administered at pretest (1 day before the intervention), posttest (1 day after the intervention), and during the follow-up test (4 weeks after the intervention). Secondary outcomes (kinematic data captured by the sensory device) from day one to day 14 of the intervention period were recorded. The outcome assessment was conducted with each participant in the pretest, posttest, and follow-up evaluations. The assessments were carried out by the same rater each time, who was unaware of the training for the participants. The raters were also unaware of the groups to which the participants were assigned. Each evaluation session lasted for 1 hrs.

The primary outcome measures included: (1) the Fugl-Meyer Assessment for upper extremity (FMA-UE); (2) the Action Research Arm Test (ARAT); and (3) the Motor Activity Log (MAL) assessment of arm use. The FMA-UE is a performance-based index used to measure poststroke hemiplegic patients’ arm and hand impairments as well as the ability to produce upper limb movements and simultaneous synergistic patterns; it consists of a three-point scale with a total possible score of 66 (Duncan et al., 1983). A higher score indicates better upper extremity motor function. FMA-UE scores are categorized into an upper extremity subscore and a hand subscore. The ARAT evaluates poststroke upper extremity functions through 19 items that are grouped into four subtests: grasping, gripping, pinching, and gross movement (Yozbatiran et al., 2008). Each test is scored on an ordinal four-point scale ranging from zero to three. A higher score represents better upper extremity function. The total possible score for all 19 items is 57. The ARAT has shown good validity and reliability in a local study (Ng et al., 2008). The MAL is a reliable self-rated questionnaire assessing the extent of the actual use of the impaired arm across 30 activities of daily living (ADLs). Ratings are assigned to each of the 30 activities according to two subscales: (1) the quality of movement (QOM); and (2) the amount of use (AOU). These two aspects are rated on a six-point scale from zero to five, in which a higher score implies higher quality and amount of use, respectively (Uswatte et al., 2006). Good psychometric properties of the local MAL version have been shown (Ng et al., 2008).

The secondary outcome measures included kinematic data recorded by the built-in accelerometer in the wristwatch (Uswatte et al., 2006). These data included the average of the total number of movements each day along the X, Y, and Z directions within the 3-hr wearing period. The range of frequency in movement detection is set at 5 Hz, captured every 2 s. This is a normal frequency range for arm movement for most daily functional tasks, while reducing a lot of unwanted minor movements, such as hand tremors, being detected. Therefore, it provides a more accurate measurement of arm movements in daily activities (Wei et al., 2019b). The outcomes of the 14-day average movement frequency, as well as the gain scores of average movement frequency between the first 3 days and the last 3 days were used for comparison.

### 2.5. Statistical analysis

IBM SPSS Statistics was used to conduct the data analysis. A Friedman test was used to evaluate the outcomes, which are the within-group differences in the open-loop and closed-loop groups, respectively, in regard to hand functions and the use of the upper limb across the three measurement time points, that is, before and after training, and at the follow-up sessions 4 weeks after training. The Wilcoxon signed-rank test was used to determine the within-group differences for the average movement frequency for the first 3 days and the last 3 days. Quade nonparametric ANCOVA with age, onset of stroke to treatment, and participants’ levels of upper limb functioning (FTHUE levels) as covariates was used to find out the between-group differences for gain score (1) (posttest minus pretest) and gain score (2) (follow-up minus pretest). The level of significance was set at *p* ≤ .05.

## 3. Results

Table 1 lists the baseline demographic characteristics of the participants. There were no significant differences between the two groups in the baselines. Table 2 lists the results of the functional outcome measures between groups at the pretest, posttest, and follow-up time points, as well as the kinematic measurement results.

**Table 1.** Baseline demographics of study participants

Characteristics	All (*n* = 16)	Closed-loop SCW-V2 (*n* = 8)	Open loop SCW-V2 (*n* = 8)	*P*
Gender (M/F)	9 (56.3)/ 7 (43.8)	3 (37.5)/ 5 (62.5)	6 (75.0)/2 (25.0)	.617
Age (y)	61 ± 9.0	58.1 ± 10.6	63.9 ± 5.8	.269
Onset of stroke to treatment (m)	60.8 ± 521.2	49.6 ± 20.5	71.9 ± 64.3	.674
Type of stroke				.317
Hemorrhagic	6 (37.5)	4 (50.0)	2 (25.0)	
Ischemic	10 (62.5)	4 (50.0)	6 (75.0)	
Right-hand dominance	15 (93.8)	7 (87.5)	8 (100)	.302
Hemiparetic side (left/right)	7 (43.8)/ 9 (56.3)	3 (37.5)/ 5 (62.5)	4 (50.0)/4 (50.0)	.614
FTHUE levels				0.99
Lower functioning	8 (50.0)	4 (50.0)	4 (50.0)	
Higher functioning	8 (50.0)	4 (50.0)	4 (50.0)	
MAS score				.418
0	2 (12.5)	1 (12.5)	1 (12.5)	
1	6 (37.5)	2 (25.0)	4 (50.0)	
1+	8 (50.0)	5 (62.5)	3 (37.5)	
Self-perceived % remained in light touch sensation				.056
0–30	3 (18.75)	2 (25.0)	1 (12.5)	
40–70	3 (18.75)	3 (37.5)	0 (0)	
80–100	10 (62.5)	3 (37.5)	7 (87.5)	
Pre-treatment assessment result as baseline score				
ARAT	31.81 ± 17.63	34 ± 15.75	29.63 ± 20.18	.636
FMA-UE	44.44 ± 14.03	48.13 ± 9.39	40.75 ± 17.40	.494
MAL-AOU	1.75 ± 1.28	1.7 ± 1.11	1.76 ± 1.48	.834
MAL-QOM	1.74 ± 1.20	1.85 ± 1.15	1.62 ± 1.33	.495
First day movement frequency	431.78 ± 146.30	390.56 ± 175.61	473.01 ± 105.56	.293

*Note:* Values expressed as *n* (%) or mean ± SD.

Abbreviations: MAS refers to the Modified Ashworth Scale; self-perceived % remained in light touch sensation refers to the percentage of light touch sensation that the participant feels in their affected side in comparison to the non-affected side.

**Table 2.** Comparison of outcome measures within-group and between-group at pretest, posttest, and follow-up

Outcome measures	Pretest (pre)		Posttest (post)		Follow-up (FU)		*x* ^2^/*z*		Within-group^1^		Between-group^2^	
	Open-loop (*n* = 8)	Closed-loop (*n* = 8)	Open-loop (*n* = 8)	Closed-loop (*n* = 8)	Open-loop (*n* = 8)	Closed-loop (*n* = 8)	Open-loop	Closed-loop	Open-loop *p*	Closed-loop *p*	Gain score (1) (Post-pre) *p*	Gain score (2) (FU-pre) *p*
ARAT	29.63 ± 20.18	34.00 ± 15.75	32.00 ± 19.88	37.75 ± 15.31	31.63 ± 20.54	38.00 ± 15.30	7.24	10.38	0.027*	0.006*	0.232	0.050*
FMA-UE (total)	40.75 ± 17.40	48.13 ± 9.39	42.75 ± 17.20	51.38 ± 7.60	43.25 ± 17.71	51.38 ± 7.93	5.55	7.58	0.062	0.023*	0.251	0.358
UL subscore	28.38 ± 7.23	31.13 ± 1.13	29.00 ± 6.85	32.75 ± 1.83	28.88 ± 6.94	32.00 ± 2.14	1.17	7.30	0.420	0.026*	0.110	0.535
Hand subscore	12.37 ± 10.74	17.00 ± 8.75	13.75 ± 11.03	18.63 ± 6.76	14.38 ± 11.25	19.38 ± 7.42	7.05	2.33	0.029*	0.311	0.917	0.530
MAL–AOU	1.79 ± 1.50	1.71 ± 1.12	2.17 ± 1.71	2.42 ± 0.97	2.04 ± 1.62	2.48 ± 0.93	6.50	9.87	0.039*	0.007*	0.137	0.006*
MAL–QOM	1.62 ± 1.33	1.85 ± 1.15	2.05 ± 1.62	2.52 ± 1.05	2.22 ± 1.51	2.69 ± 1.09	12.3	14.0	0.002*	0.001*	0.131	0.030*
^¶^Average frequency of movements	352 ± 98.38	496 ± 140.91	266 ± 107.17	495 ± 142.50	NA	NA	−1.68 −0.42		0.093 0.674		0.432	NA

*Note:* Values are shown as mean ± SD. Pretest indicates 1 day before intervention; posttest indicates 1 day after intervention; and follow-up indicates 4 weeks after intervention; *p ≤ 0.05; ^1^Friedman test (***x*** ^**2**^**)** or Wilcoxon signed-rank test (*z*) for ^¶^average movement frequency for the first 3 days and the last 3 days; ^2^Quade nonparametric ANCOVA across the measurement time points with age, onset of stroke to treatment, and FTHUE levels as covariates.

### 3.1. Effects on upper extremity motor performance

Upper-extremity motor performance and functions were evaluated using the FMA-UE and ARAT, respectively. The closed-loop RTM group showed significant improvements among pretest, posttest, and follow-up evaluations in regard to FMA-UE total score (*x* ^2^ = 7.58, *p* = .023) and its upper limb subscore (*x* ^2^ = 1.17, p = .026), but significant within-group differences were found in the open-loop group for the hand subscore (*x* ^2^ = 7.05, *p* = .029); there were no significant differences in the FMA-UE total scores and subscores between groups. The ARAT score showed significant improvement within both closed-loop (*x* ^2^ = 10.38, *p* = .006) and open-loop groups (*x* ^2^ = 7.24, *p =* .027). There were significant between-group differences for gain score (2) in the ARAT (*p* = .050). The improvement over the ARAT across the three measurement time points in the closed-loop group was higher than that of the open-loop group (Table 2).

### 3.2. Effects on actual arm use

**Figure 3.**
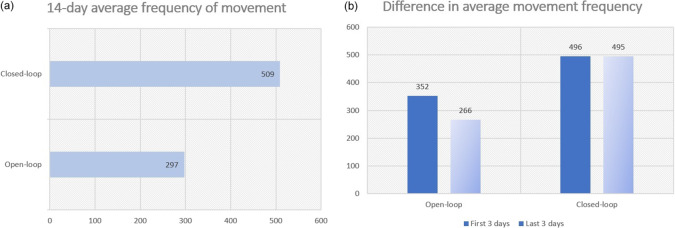
Line graphs of the kinetic measures between groups.

Actual arm use was objectively evaluated using the kinematic data recorded by the built-in logger in the cueing device. We found that participants wearing closed-loop devices moved their upper limbs significantly more than those who wore open-loop devices, as indicated by the significant difference between the open-loop and closed-loop groups in the average frequency of movements between the first 3 days and that of the last 3 days (F = 14.91, *p* = .002). Figure 3 shows that the closed-loop group increased more in the 14-day average frequency of movement and a higher gain score in average movement frequency between the first 3 days and the last 3 days as compared to the open-loop group (Figure 3b).

Actual arm use was subjectively assessed using both the MAL-QOM and the MAL-AOU. There were significant differences within-groups in the MAL–AOU (Open-loop *x* ^2^ = 6.5, *p =* .039; Closed-loop *x* ^2^ = 9.87, *p =* .007) and MAL–QOM (Open-loop *x* ^2^ = 12.3, *p =* .002; Closed-loop *x* ^2^ = 14.0, *p =* .001) across the three measurement time points. Significant between-groups differences were found in gain score (2) of MAL-QOM and MAL-AOU (*p* = .006 and *p* = .030, respectively) (Table 2).

### 3.3. Discussion

Both open-loop and closed-loop RTM training led to significant improvements in the hand functions measured using ARAT and the actual arm use measured using MAL, as well as the frequency of movements. The closed-loop group improved more significantly than the open-loop group in terms of hand functions and the actual arm use as self-reported in the MAL, and the average movement frequency in the closed-loop group was higher than that in the open-loop group. However, the improvement in the closed-loop group could not be seen in the upper limb motor performance measured using the FMA.

The closed-loop wearable device is more useful as it can act as an internal reminder, which facilitates the habit formation of participants in using their hemiparetic arm more frequently in daily life. It is likely that the open-loop group became reliant on the cue to initiate exercise and ceased exercising during the follow-up, whereas the closed-loop group was able to carry forward the self-initiated habit to the follow-up period and continued to improve. Participants wearing a closed-loop device will be reminded constantly if they do not use their hemiparetic arm within the fixed time interval of 2–5 mins. To reduce the frequency of the vibration elicited by the device, participants in the closed-loop group tended to increase their use of the hemiparetic limb in daily activities. This results in avoidance conditioning and hence reinforces participants moving their paretic arms more frequently. Since closed-loop devices encourage self-initiated movement intrinsically, this may eventually help build up their movement habits. The use of wearables is very important to the rehabilitation of stroke patients, as it favors the retention of treatment effects outside therapy sessions. The nonuse condition could be ameliorated to a larger extent. This phenomenon induced by the closed-loop RTM, appears to adhere to the neuroplasticity principles of “use it or lose it” and “use it and improve it” as well as “sufficient repetition” and “sufficient training intensity,” leading to the induction of plasticity (Kleim and Jones, 2008). In contrast with the open-loop group, participants in the closed-loop group could not foresee when sensory cues would be elicited from the wristwatch. To avoid the undesired cue elicitation, participants in the closed-loop group tended to have higher movement frequency associated with the movements. More practice of repetitive tasks, particularly under the supervision of trained rehabilitation professionals, can promote the generalization of task components even when the components are scaled to novel amplitudes or durations. The open-loop wearable, on the other hand, also contributes to improvements in upper limb function and movement frequency, since it provides external signals regularly, serving as passive reminders to participants, but might not be as strong as the closed-loop function as seen in this study. The findings are consistent with the recent clinical trial using wearable-based intervention for tele-rehabilitation to encourage self-directed repetitive arm movements to boost upper extremity recovery in chronic stroke patients in the community (Toh et al., 2025). Open-loop wearables emit signals regularly to remind participants to move their paretic limb, regardless of their movement frequency, while any usage of the upper limb in daily activities detected by the closed-loop devices causes the device to start counting down again and postpone the elicitation of signals. As a result, participants in the closed-loop group may receive fewer reminders than those in the open-loop group.

Unfortunately, the improvement in the closed-loop group could not be translated to upper limb motor control as measured using the FMA-UE in this study. As upper limb and hand motor control are far more complex than exercise, participants in the closed-loop group still made improvements in arm and hand control, but the improvement is not significant as compared to the open-loop group.

We observed some phenomena that could indicate possible directions for future rehabilitations. First, RTM treatment is more beneficial to participants with a high level of functioning with learned nonuse. Before receiving treatment, they mainly used the less affected arm to perform daily tasks. Another phenomenon is that participants commonly had a higher frequency of movement in the first 3 days for both groups. They had higher motivation and interest at the beginning. When there was no immediately observable improvement, they were less motivated and became fatigued. As a result, they moved less than at the start of the intervention period. We found that only the closed-loop group maintained a higher frequency of movement, since they developed a habit of moving their hemiparetic arms more frequently to avoid “punishment.” Approaches to reinforcement and encouragement to improve patients’ compliance with the wearing regime in order to facilitate treatment outcomes warrant further studies.

## 4. Limitations

There are several limitations to this study. First, the generalizability of this proof-of-concept study is limited due to its small sample size. Therefore, replication of the study with a larger sample size would be beneficial. Second, the RTM devices used in this study lack the sensitivity necessary to detect finger movements, particularly those participants with higher upper extremity functioning. Third, we have not surveyed the participants on the usability of the wearables and their acceptance of the treatment procedures. Moreover, the effect of individual variations in the participants’ daily routines on the frequency of using their hemiparetic arms should be taken into consideration. In addition, the method of using activity counts from wrist-worn accelerometers has been challenged by the high interindividual and intraindividual variability of movement patterns for persons after stroke, as well as the sensitivities of the sensors, which depends very much on the optimal thresholds (Pohl et al., 2022) and that they do not correlate well with daily hand use (Rast and Labruyère, 2022; Wei et al., 2019b). Future studies should be done on developing an accurate algorithm for wrist-worn accelerometry for clinical purposes (Lum et al., 2020).

## 5. Conclusions

This proof-of-concept study supports our hypothesis that the closed-loop RTM is more effective than the open-loop RTM training in improving the movement frequency and actual arm use, as well as overcoming learned nonuse of the hemiparetic limbs in participants with chronic stroke. The closed-loop RTM concept has demonstrated potential efficacy in improving impairment of the hemiparetic upper extremity through tele-rehabilitation for patients with stroke. In the future, the closed-loop RTM mechanism can be incorporated in the wearables to enhance the treatment efficacy.

## Data Availability

The data that support the findings of this study are available on request from the corresponding author [KNKF]. The data are not publicly available due to ethical restrictions; they contain information that could compromise the privacy of research participants.

## References

[r1] Antony JW, Ngo HV, Bergmann TO and Rasch B (2022) Real‐time, closed‐loop, or open‐loop stimulation? Navigating a terminological jungle. Journal of Sleep Research 31(6), e13755. 10.1111/jsr.13755. 36285430

[r2] Bai Z and Fong KNK (2020) Remind-to-move” treatment enhanced activation of the primary motor cortex in patients with stroke. Brain Topography 33(2), 275–283. 10.1007/s10548-020-00756-7. 32056031

[r3] Bailey RR, Klaesner JW and Lang CE (2015) Quantifying real-world upper-limb activity in nondisabled adults and adults with chronic stroke. Neurorehabilitation and Neural Repair 29(10), 969–978. 10.1177/1545968315583720. 25896988 PMC4615281

[r4] Bohannon RW and Smith MB (1987) Interrater reliability of a modified Ashworth scale of muscle spasticity. Physical Therapy 67, 206–207. 3809245 10.1093/ptj/67.2.206

[r5] Dong VA, Fong KNK, Chen Y, Tseng SSW and Wong LMS (2017) Remind-to-move’ treatment versus constraint-induced movement therapy for children with hemiplegic cerebral palsy: A randomized controlled trial. Developmental Medicine & Child Neurology 59(2), 160–167. 27503605 10.1111/dmcn.13216

[r33] Duncan PW, Propst M and Nelson SG (1983) Reliability of the Fugl-Meyer assessment of sensorimotor recovery following cerebrovascular accident. Physical Therapy 3(10), 1606–1610. 10.1093/ptj/63.10.1606. 6622535

[r6] Faria-Fortini I, Michaelsen SM, Cassiano JG and Teixeira-Salmela LF (2011) Upper extremity function in stroke subjects: Relationships between the international classification of functioning, disability, and health domains. Journal of Hand Therapy 24(3), 257–264. 21420279 10.1016/j.jht.2011.01.002

[r7] Fong K, Ng B, Chan D, Chan E, Ma D, Au B, Chiu V, Chang A, Wan K, Chan A and Chan V (2004) Development of the Hong Kong Version of the Functional Test for the Hemiplegic Upper Extremity (FTHUE-HK). *Hong Kong Journal of Occupational Therapy* 14(1), 21–29. 10.1016/S1569-1861(09)70025-7

[r8] Fong KN, Lo PC, Yu YS, Cheuk CK, Tsang TH, Po AS and Chan CC (2011) Effects of sensory cueing on voluntary arm use for patients with chronic stroke: A preliminary study. Archives of Physical Medicine and Rehabilitation 92(1), 15–23. 10.1016/j.apmr.2010.09.014. 21187201

[r9] Fong KNK, Yang NY, Chan MK, Chan DY, Lau AF, Chan DY, Cheung JT, Cheung HK, Chung RC and Chan CC (2013) Combined effects of sensory cueing and limb activation on unilateral neglect in subacute left hemiplegic stroke patients: A randomized controlled pilot study. Clinical Rehabilitation 27(7), 628–637. 10.1177/0269215512471959. 23405025

[r10] Fritz SL, Chiu Y-P, Malcolm MP, Patterson TS and Light KE (2005) Feasibility of electromyography-triggered neuromuscular stimulation as an adjunct to constraint-induced movement therapy. Physical Therapy 85(5), 428–442. 10.1093/ptj/85.5.428. 15842191

[r12] Grefkes C and Fink GR (2020) Recovery from stroke: Current concepts and future perspectives. Neurological Research and Practice 2(1), 17–17. 10.1186/s42466-020-00060-6. 33324923 PMC7650109

[r13] Ingram LA, Butler AA, Brodie MA, Lord SR and Gandevia SC (2021) Quantifying upper limb motor impairment in chronic stroke: A physiological profiling approach. Journal of Applied Physiology 131(3), 949–965. 10.1152/japplphysiol.00078.2021. 34264125

[r14] Kleim JA and Jones TA (2008) Principles of experience-dependent neural plasticity: Implications for rehabilitation after brain damage. Journal of Speech, Language, and Hearing Research 51(1), S225–S239. 10.1044/1092-4388(2008/018). 18230848

[r15] Kwakkel G, Kollen BJ, Van der Grond JV and Prevo AJ (2003) Probability of regaining dexterity in the flaccid upper limb: Impact of severity of paresis and time since onset in acute stroke. Stroke 34(9), 2181–2186. 10.1161/01.STR.0000087172.16305.CD. 12907818

[r16] Lum PS, Shu L, Bochniewicz EM, Tran T, Chang LC, Barth J and Dromerick AW (2020) Improving accelerometry-based measurement of functional use of the upper extremity after stroke: Machine learning versus counts threshold method. Neurorehabilitation and Neural Repair 34(12), 1078–1087. 33150830 10.1177/1545968320962483 PMC7704838

[r18] Mirza KB, Golden CT, Nikolic K and Toumazou C (2019) Closed-loop implantable therapeutic neuromodulation systems based on neurochemical monitoring. Frontiers in Neuroscience 13, 808. 10.3389/fnins.2019.00808. 31481864 PMC6710388

[r19] Ng AK, Leung DP and Fong KNK (2008) Clinical utility of the action research arm test, the Wolf Motor function test and the motor activity log for hemiparetic upper extremity functions after stroke: A pilot study. Hong Kong Journal of Occupational Therapy 18(1), 20–27. 10.1016/S1569-1861(08)70009-3.

[r20] Pohl J, Ryser A, Veerbeek JM, Verheyden G, Vogt JE, Luft AR and Awai Easthope C (2022) Classification of functional and non-functional arm use by inertial measurement units in individuals with upper limb impairment after stroke. Frontiers in Physiology 13, 952757. 10.3389/fphys.2022.952757. 36246133 PMC9554104

[r21] Raghavan PP (2015) Upper limb motor impairment post stroke. Physical Medicine and Rehabilitation Clinics of North America 26(4), 599–610. 10.1016/j.pmr.2015.06.008. 26522900 PMC4844548

[r22] Rast FM and Labruyère R (2022) Concurrent validity of different sensor-based measures: Activity counts do not reflect functional hand use in children and adolescents with upper limb impairments. Archives of Physical Medicine and Rehabilitation 103(10), 1967–1974. 35439522 10.1016/j.apmr.2022.03.021

[r23] Rodgers H, Bosomworth H, Krebs HI, van Wijck F, Howel D, Wilson N, Aird L, Alvarado N, Andole S, Cohen DL, Dawson J, Fernandez-Garcia C, Finch T, Ford GA, Francis R, Hogg S, Hughes N, Price CI, Ternent L, et al. (2019) Robot assisted training for the upper limb after stroke (RATULS): A multicentre randomised controlled trial. Lancet 394, 51–62. 10.1016/S0140-6736(19)31055-4. 31128926 PMC6620612

[r24] Taub E, Miller NE, Novack TA, Cook EW, Fleming WC, Nepomuceno CS, Connell JS and Crago JE (1993) Technique to improve chronic motor deficit after stroke. Archives of Physical Medicine and Rehabilitation 74, 347–354. 8466415

[r25] Taub E, Uswatte G, Mark VW and Morris DM (2006) The learned nonuse phenomenon: Implications for rehabilitation. Europa Medicophysica 42(3), 241–256. 17039223

[r26] Toh SFM, Cruz Gonzalez P and Fong KNK (2023a) Usability of a wearable device for home-based upper limb telerehabilitation in persons with stroke: A mixed-methods study. Digital Health 9, 1–15. 10.1177/20552076231153737. PMC9909064 36776407

[r27] Toh SFM, Fong KNK, Gonzalez PC and Tang YM (2023b) Application of home-based wearable technologies in physical rehabilitation for stroke: A scoping review. IEEE Transactions on Neural Systems and Rehabilitation Engineering 31, 1614–1623. 10.1109/TNSRE.2023.3252880. 37028029

[r28] Toh FM, Lam WWT, Cruz Gonzalez P and Fong KNK (2025) Effects of a wearable-based intervention on the hemiparetic upper limb in persons with stroke: A randomized controlled trial. Neurorehabilitation and Neural Repair 39(1), 31–46. 10.1177/15459683241283412. 39328083

[r29] Uswatte G, Taub E, Morris D, Light K and Thompson P (2006) The motor activity log-28: Assessing daily use of the hemiparetic arm after stroke. Neurology 67(7), 1189–1194. 10.1212/01.wnl.0000238164.90657.c2. 17030751

[r30] Wei WXJ, Fong KNK, Chung RCK, Cheung HKY and Chow ESL (2019a) “Remind-to-move” for promoting upper extremity recovery using wearable devices in subacute stroke: A multicenter randomized controlled study. IEEE Transactions on Neural Systems & Rehabilitation Engineering 27(1), 51–59. 30475722 10.1109/TNSRE.2018.2882235

[r31] Wei WXJ, Fong KNK, Chung RCK, Myint JMWW, Cheung HKY and Chow ESL (2019b) Utility of a unilateral accelerometer for monitoring upper extremity use in subacute stroke patients after discharge from hospital. Assistive Technology 31(4), 193–198. 10.1080/10400435.2017.1414085. 29215963

[r32] World Health Organization (2006) WHO STEPS stroke manual. Available at https://apps.who.int/iris/bitstream/handle/10665/43420/9241594047_eng.pdf (accessed 24 September 2024)

[r34] Yozbatiran N, Der-Yeghiaian L and Cramer SC (2008) A standardized approach to performing the Action Research Arm Test. Neurorehabilitation and Neural Repair 22(1), 78–90. 10.1177/1545968307305353. 17704352

